# PRIMA-1^MET^ induces death in soft-tissue sarcomas cell independent of p53

**DOI:** 10.1186/s12885-015-1667-1

**Published:** 2015-10-13

**Authors:** Thomas Grellety, Audrey Laroche-Clary, Vanessa Chaire, Pauline Lagarde, Frédéric Chibon, Agnes Neuville, Antoine Italiano

**Affiliations:** 1 National Institute of Health and Medical research, INSERM U916, Institut Bergonié, Bordeaux, France; 2 University of Bordeaux, Bordeaux, France; 3 Department of Medical Oncology, Institut Bergonié, Bordeaux, France; 4 Department of Pathology, Institut Bergonié, Bordeaux, France

**Keywords:** Soft tissue sarcoma, Apoptosis, Oxidative stress, PRIMA-1^MET^, p53

## Abstract

**Background:**

The aim of this study was to explore the efficacy and define mechanisms of action of PRIMA-1^MET^ as a TP53 targeted therapy in soft-tissue sarcoma (STS) cells.

**Methods:**

We investigated effects of PRIMA-1^MET^ on apoptosis, cell cycle, and induction of oxidative stress and autophagy in a panel of 6 STS cell lines with different TP53 status.

**Results:**

Cell viability reduction by PRIMA-1^MET^ was significantly observed in 5 out of 6 STS cell lines. We found that PRIMA-1^MET^ was capable to induce cell death not only in STS cells harboring mutated TP53 but also in TP53-null STS cells demonstrating that PRIMA-1^MET^ can induce cell death independently of TP53 in STS cells. We identified an important role of reactive oxygen species (ROS), involved in PRIMA-1^MET^ toxicity in STS cells leading to a caspase-independent cell death. ROS toxicity was associated with autophagy induction or JNK pathway activation which represented potential mechanisms of cell death induced by PRIMA-1^MET^ in STS.

**Conclusions:**

PRIMA-1^MET^ anti-tumor activity in STS partly results from off-target effects involving ROS toxicity and do not deserve further development as a TP53-targeted therapy in this setting.

## Background

Well-planned wide surgical excision complemented by adjuvant radiotherapy in cases of large (>5 cm) and/or deep tumors is the standard loco-regional treatment for soft-tissue sarcoma (STS) patients [1, 2]. However, despite optimal local treatment, 30 to 40 % of patients will develop metastatic disease [3]. Doxorubicin is considered as the standard first-line therapy for patients with advanced STS and median overall survival is only 12–18 months [4]. New therapies are therefore needed in this setting.

TP53 is a transcription factor also called “guardian of genome”, activated under stress conditions, leading to apoptosis induction and cell cycle arrest [5]. Our research group has previously shown that the p53 pathway is commonly dysregulated in STS [6] with mutations found in more than 50 % of STS with complex genomics profiles. Mutations affecting TP53 cause misfolded protein and/or lack of DNA interaction capacity [7], (http://p53.iarc.fr/) with the result of lack of tumor suppressor function and more recently discovered an oncogenic gain function [7]. More than 80 % of the mutation occurs in the DNA-binding domain of the gene preventing correct transcription activity [7]. Based on its crucial role in carcinogenesis and the frequent dysregulation of the p53 pathway in cancer, TP53 represent an appealing target for mechanism-driven anticancer drug discovery.

Nutlins, which prevent p53 proteasomal degradation via MDM2 interaction [8] has proven some preclinical efficacy and is now under early clinical phase development. In 2002, Bykov et al. [9] isolated from the low molecular weight compound library of the National Cancer Institute, a compound that can restore wild-type function to mutant p53, called PRIMA-1 for *P53 Reactivation and Induction of Massive Apoptosis*. The described molecular mechanism of action is based on the conversion of PRIMA-1 into compounds that form adducts with thiols in mutant TP53 allowing transcription activation [10]. Demonstration of the *in vitro* and *in vivo* activity of PRIMA-1 or its methylated form, PRIMA-1^MET^, in terms of apoptosis induction [11–13] and cell cycle arrest [12, 13] has been reported in different tumor models. There is no data regarding the activity of PRIMA-1^MET^ in STS.

The aim of our study was to obtain preliminary proof of efficacy of PRIMA-1^MET^ in STS cell lines and to assess its specific mechanism of action regarding TP53.

## Methods

### Cells

The STS cell lines, IB130 (pleiomorphic liposarcoma/ mutant TP53 exon 8, P278L), IB134 (uterine leiomyosarcoma/mutant TP53 exon 6, S215R), IB136 (soft-tissue leiomyosarcoma, null TP53), IB117 (myxofibrosarcoma, null TP53), IB138 (soft-tissue leiomyosarcoma/mutant TP53 exon 5, V143M), and IB139 (soft tissue leiomyosarcoma, wild-type TP53) used in this study have been derived from human surgical specimen of STS in the laboratory of Pr. Jean-Michel Coindre and Dr Frédéric Chibon (Institut Berognié, Bordeaux, France) and after having obtained patient consent. For all the cell lines, TP53 status was assessed by Sanger sequencing, array-comparative genomic hybridization and western blotting (protocols are available on request). Colon carcinoma cell lines used were HCT-116 (wild type TP53) and HT-29 (mutant TP53 exon 8, R273H) purchase from the NCI (http://discover.nci.nih.gov/cellminer/). All cell lines were cultured in complete RPMI 1640 (Sigma Life Technologies, Saint Louis, MO) with 10 % Fetal calf serum, Penicillin/Streptomycin 1 %, and Normocin 0.2 %.

### Reagents

PRIMA-1^MET^ and Staurosporin were purchased from Santa Cruz Biotechnology INC (Heidelberg, Germany). PRIMA-1^MET^ was stored at −20 °C and diluted in water. Chloroquine Diphosphate salt and N-acetyl-L-cysteine were purchased from Sigma Life Science (Saint Louis, MO).

### Cell viability

Three thousand cells were seeded in 96-well plates for 24 hr and treated with a range of increasing concentrations of PRIMA-1^MET^ for 24 hr to 96 hr. Methyl Thiazolyl Tétrazolium (MTT, Sigma Aldrich, St Quentin Fallavier, France) was applied for 3 hours before being dissolved in dimethylsufoxyde (final concentration: 0.5 mg/mL). Quantity of produced formazan was measured by spectrophotometry. Absorbance was measured at 570 nm with a reference at 630 nm. Analysis was done by using the KC4 software (Kinetical for Windows V.3.4) and IC50 was calculated with GraphPad Prism version 5.00 for Windows (GraphPad Software, La Jolla California USA, www.graphpad.com).

### Fluorescent cell sorting analysis (FACS)

Apoptosis and cell cycle were evaluated using Fluorescent Activating Cell Sorting (FACS) analysis. For mitochondrial membrane depolarization studies, 3000 cells were seeded in 96-well plates for 24 hours and treated with a range of increasing concentrations of PRIMA-1^MET^ for 72 hours, then incubated for 30 min with Tetramethylrhodamine Methyl Ester (TMRM). P-glycoprotein drug efflux pump was blocked using Verapamil (Sigma-Aldrich, St Quentin Fallavier, France). For activated caspases 3 and 7 detection, 5.10^5^ cells were seeded in 6-wells plates for 24 hours, treated with increasing doses of PRIMA-1^MET^ for 72 hours and 96 hours, respectively. Cells were harvested and exposed to FLICA 1X as described by the supplier (FAM-FLICA™ Kit, ImmunoChemistry Technologies, Bloomington, USA) for 1 hour. For apopotosis/necrosis assay, 1.10^6^ cells were seeded in 6-wells plates for 72 hours, then treated and exposed to FITC-Annexin and propidium iodide (PI) according the manufacturer’s protocol (BD Biosciences, Erembodegem, Belgium). This allowed distinguishing annexin V positive cells in early apoptosis, versus annexin V and PI positives cells in late apoptosis or necrosis. For cell cycle analysis, 1.10^5^ cells were seeded in 6-wells plates and after 24 hours cells were treated with PRIMA-1^MET^ for 48 hours to 96 hours. Cells were then fixed and permeabilized in absolute ethanol with PBS over-night at −20 °C, then rinsed and incubated with RNase and Propidium Iodide (50 μg/mL) (Sigma Aldrich, St Quentin Fallavier, France). For ROS production assay, 6000 cells were seeded in 96-well plates for 24 hours and treated with PRIMA-1^MET^ for 96 hours, then incubated for 30 min with 2',7'-Dichlorofluorescin diacetate (DCFDA) 20 μM (Abcam, Cambridge, MA, USA). We performed flow cytometry analysis using FL1 for TMRM, FAM-FLICA, Annexin-V and DCFDA, whereas FL2 was used for Propidium iodide. Flow cytometry (FACSCalibur; BD Biosciences, San Jose, USA) data were analyzed with FlowJo v.7.6.3. software.

### Immunoblot analysis

The physical lysis « Ultimate freeze-thaw lysis for mammalian cells » protocol was used for non-phosphorylated protein extraction [14]. For autophagy and phosphorylated protein, Radio-ImmunoPrecipitation Assay (RIPA) lysis buffer protocol [15] was used. Total proteins (30-60 μg) were separated by 10 or 12 % SDS-PAGE and transferred on Polyvinyl Difluoride (PVDF) membrane. The following antibodies were purchased from Cell Signaling Technology (Danvers, USA): anti-BAX (1:500), anti-PUMA (1:1000), anti-JNK (1:1000) and anti-phospho JNK monoclonal antibodies (1:1000). Anti-GAPDH (1:200) and anti-p53 (1:1000) monoclonal antibodies were purchased from Santa Cruz Biotechnology (Heidelberg, Germany). The anti-p21 monoclonal antibody (1:33) was purchased from Calbiochem (San Diego, USA). The anti-LC3IIB monoclonal antibody (1:1000) was purchased from Sigma Aldrich (Saint Louis, USA) and the anti-PARP monoclonal antibody (1:1000) was purchased from EnzoLifeSciences (Farmingdale, USA). Secondary antibodies anti-Mouse IgG and anti-Rabbit IgG (1:5000) were purchased from Ge Healthcare (Buckinghamshire, United Kingdom). Proteins were detected (Fusion Fx7, Fisher Bioblock Scientific, Waltham, MA, USA) by using enhanced chemiluminescent substrate for horseradish peroxidase (HRP) (Immobilon™ Western, Millipore Corporation). Each membrane was reused 3 times after desaturation in glycin (6.6 mol/L) buffer pH = 2 at 56 °C for 30 min. Semi-quantitative analysis was realized with ImageJ 1.45 s software (Rasband, W.S., ImageJ, U. S. National Institutes of Health, Bethesda, Maryland, USA, http://imagej.nih.gov/ij/, 1997–2014).

### Confocal microscopy

Cells were seeded on coverslips and treated with PRIMA-1^MET^ for 72 hours. Slides were then washed twice with PBS, fixed in formaldehyde 4 % and incubated with anti-LC3IIB monoclonal antibody (Sigma Aldrich, Saint Louis, USA) overnight, and then with a goat anti-rabbit Alexa fluor 488 antibody (Invitrogen, Paisley, United Kingdom). Slides were then counterstained by 4,6-diamidino-2-phenylindole (DAPI).

## Results

### Prima-1^MET^ reduces STS cells viability independently of p53 status

**Fig. 1:**
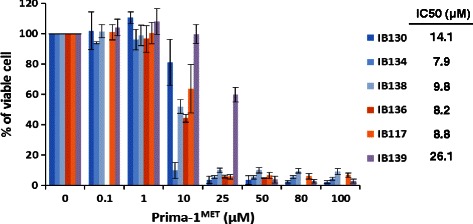
Growth suppression effect of PRIMA-1^MET^. Effect were assessed by MTT using different concentrations in each of the 6 indicated STS cell lines, with respective IC50 values, *n* = 3-4, error bars show SEM

We observed significant and similar sensitivity in term of viability reduction in the TP53 mutated (MT) STS cell lines (IB130, IB134, IB138) and in the TP53 null cell lines (IB136, IB117) after exposure to PRIMA-1^MET^ for 96 hours (Fig. 1). PRIMA-1^MET^ induced more growth inhibition in these cell lines than in the TP53 wild-type (WT) cell line (IB139). Mutated STS cell lines (IB130, IB134, IB138) and null cell lines (IB136, IB117) have equivalent IC50 (approximately 10 μM, Fig. 1), about twice less than wild type cell lines (IB139). HCT-116 (WT) and HT-29 (MT) cells were equally sensitive to PRIMA-1^MET^ (approximately 10 μM, data not shown). Those results indicate that PRIMA-1^MET^ act independently of p53 in the p53 null cell line and that PRIMA-1^MET^ is more effective in mutated than wild type STS cell lines.

### PRIMA-1^MET^ does not induce apoptosis in STS cell lines

**Fig. 2:**
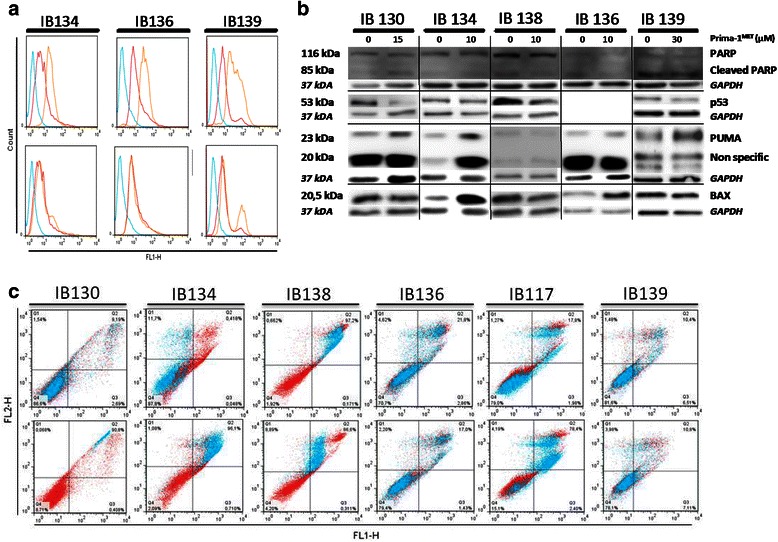
Cell death induced by PRIMA-1^MET^ on STS cell lines. **a** caspases 3 and 7 activation in cell lines, upper line represent fusion of FLICA fluorescence in cells non treated (NT) not marked (blue), NT marked (red) and treated by Staurospaurin (orange) at 1 μM (IB134), 5 μM (IB136 and IB139) for up to 72 hr. Lower line represent fusion of FLICA fluorescence in cells NT not marked (blue), NT marked (red) and treated by Prima-1MET (orange) at 60 μM (IB134) or 80 μM (IB136 and IB139) for up to 72 hr. **b** Levels of the indicated proteins evaluated by Western blot analysis after exposure for up to 72 hours to specific defined cell line IC50 PRIMA-1MET doses. **c** Cell death analyses using FITC annexin-V and propidium iodide assay. Upper line compare NT condition (red) to PRIMA-1^MET^ at IC50 cell line specific dose (blue) defined by MTT assay, lower line compare NT (red) to 80 μM of PRIMA-1^MET^ except for IB117 (50 μM) (blue)

Induction of apoptosis has been largely demonstrated as key mechanisms of action of PRIMA-1^MET^ [11–13]. By using caspase 3/7 and Annexin V assays, we found that PRIMA-1^MET^ does not induce significant apoptosis in our STS cell lines regardless of TP53 status. There were no activation of caspases 3 and 7 in mutated or deleted cell lines and a non-significant induction has only been found with high doses of PRIMA-1^MET^ in the TP53-wild type cell line IB139 (Fig. 2a). Moreover, no PARP cleavage was observed after exposure to PRIMA-1^MET^ (Fig. 2b). In addition, by using Annexin V/PI assay, no apoptosis was detected whatever the TP53 status (Fig. 2c). However, we found that PRIMA-1^MET^ increased the percentage of cell death in the three TP53-mutated and TP53-deleted cell lines compared with the wild-type cell line (IB139). No accumulation of annexin V positive cells was identified when treating HCT-116 (WT) and HT-29 (MT) with PRIMA1-^MET^ (data not shown).

### PRIMA-1^MET^ does not induce p53-dependent cell cycle arrest in STS cell lines

One of the putative mechanisms of action of PRIMA-1^MET^ is restoring p53 functions as a sequence-specific transcription factor that drives the transactivation of target genes mediating cell-cycle arrest [10, 12, 13]. Treatment of STS cells for 24, 48 or 72 hours with PRIMA-1^MET^ did not result in dose dependent cell cycle arrest and induction of P21. PRIMA-1^MET^ induced cell cycle arrest in HCT-116 cells in a dose dependent manner (data not shown). Those results combined with the lack of apoptosis suggest that PRIMA-1^MET^ effect is largely p53 independent in STS cells.

### PRIMA-1^MET^-induced STS cells death is associated with a loss of mitochondrial membrane potential (MMP) and reactive oxygen species (ROS) toxicity independently of the p53 status

**Fig. 3:**
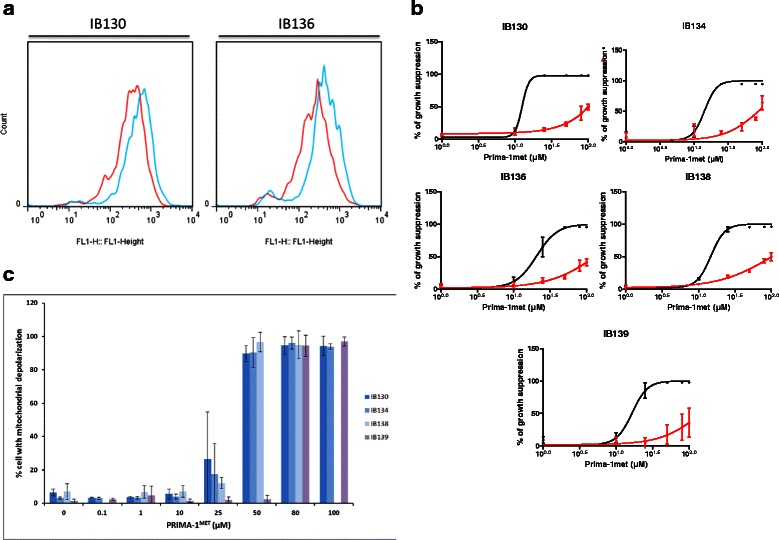
PRIMA-1^MET^ induces ROS-associated toxicity and loss of MMP. **a** ROS production estimated by DCF staining and assessed by flow cytometry, not treated in red, PRIMA-1^MET^ 30 μM (IB130) and 10 μM (IB136) for 96 h in blue. **b** ROS implication in PRIMA-1^MET^ growth suppression effects using MTT assay. Cells were treated for 96 h with range of PRIMA -1^MET^ alone (black) and combined with NAC (2,5 mM) (red). **c** Loss of MMP by cell line with TMRM fluorescent assay, *n* = 3, error bars show SEM

PRIMA-1^MET^ has been shown to exert its effect in part via inducing ROS production and therefore promoting an oxidative environment in tumor cells [10, 16, 17]. We treated our panel of STS cell lines with PRIMA-1^MET^ and we quantified ROS levels by using the fluorogenic dye DCFDA with flow cytometry. As indicated in Fig. 3a, we observed an induction of ROS by PRIMA1-^MET^ not related to p53 status. This production of ROS was accompanied with a loss of mitochondrial membrane potential (Fig. 3c). Further, we functionally analyzed the effects of blocking ROS production by N-acetyl cysteine (NAC) on PRIMA-1^MET^-induced cell growth suppression. As shown in Fig. 3b, 2.5 mM NAC treatment could completely reverse PRIMA-1^MET^-induced ROS production and suppressed PRIMA-1^MET^ anti-tumor effect in STS cell lines whatever their p53 status. Same results were found in the two colon carcinoma cell lines (data not shown).

### PRIMA-1^MET^ induces autophagy and activates JNK pathway in STS cell lines independently of TP53 status

**Fig. 4:**
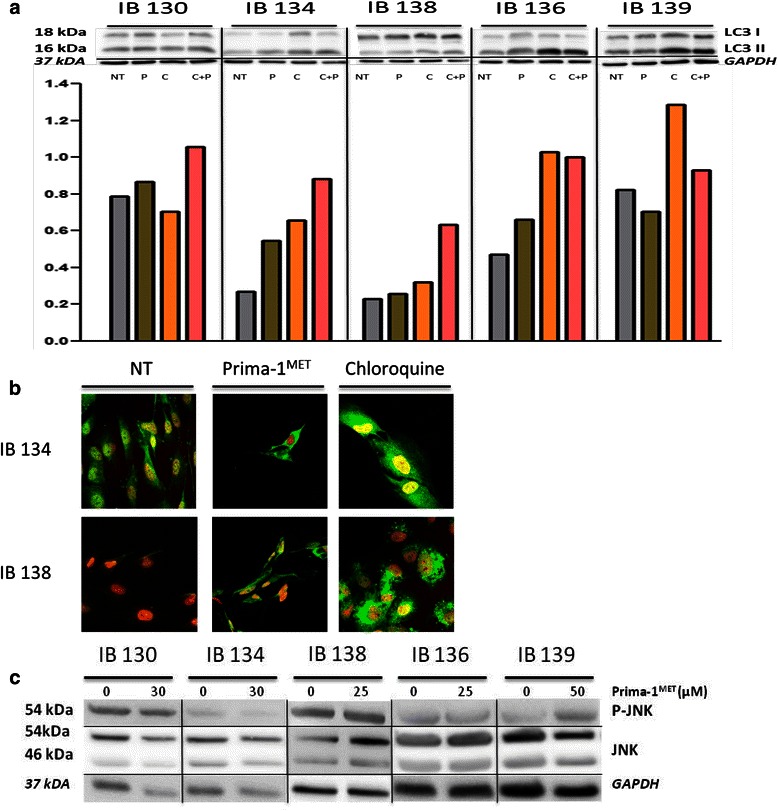
Autophagy and JNK phosphorylation induced by PRIMA-1^MET^. **a** Western blot and quantification of LC3II in each cell line after 72 hr of PRIMA-1^MET^. Chloroquine (C) was used at 20 μM, NT: Not Treated, P: PRIMA-1^MET^, C + P: Chloroquine and Prima-1^MET^. **b** IB 134 and IB 138 cells were treated and fixed for immunostaining with an antibody against LC3. Representative images from two independent experiments are shown. **c** JNK pathway activation assessed by western blot after 96 hr of PRIMA-1^MET^ exposure. For all these assays PRIMA-1^MET^ doses correspond to previously found doses responsive for loss of MMP in 50 % of treated cells assessed by TMRM assays; 30 μM for IB130 and IB134, 25 μM for IB138 and IB136 and 50 μM for IB139

The exact mechanisms involved in ROS-induced cell death are not fully understood. There are several lines of evidence indicating that oxidative stress directly induces autophagy, as a cell death mechanism. For this reason, we decided to investigate whether PRIMA-1^MET^-associated ROS toxicity was coupled with autophagy induction in STS cell lines. LC3 is specifically localized to autophagic structures. When autophagy is not activated, LC3 is localized homogeneously in the cytoplasm, while upon initiation of autophagy, it associates with the membrane of autophagosomes. Since increase in LC3-II levels or GFP-LC3 vesicles can occur not only due to increased autophagosome synthesis but also due to impaired autophagosome-lysosome fusion, we assessed LC3-II levels in the presence of chloroquine, a blocker of LC3-II degradation. Analysis of LC3-II levels by western blotting and by fluorescence microscopy allowed us to detect PRIMA-1^MET^ -induced autophagy in the two STS cell lines with TP53 mutation (IB 134 and IB 138) but not in the other ones (Fig. 4a-b). In colon carcinoma cells, autophagy was induced irrespective of TP53 status.

ROS-dependent activation of JNK has been identified as a crucial mechanism of cell death [18]. We found that PRIMA-1^MET^ induced JNK phosphorylation (p-JNK) in IB130 and IB139 cell lines but not in IB134, IB136 and IB138 (Fig. 4c). p-JNK was also induced in HT-29 cells (data not shown).

## Discussion

We report here the first study investigating PRIMA-1^MET^ activity in STS by using 6 cell lines with different TP53 status. First, we found that PRIMA-1^MET^ altered significantly cell viability in 5 out the 6 cell lines, two of them harboring a homozygous deletion of TP53. This result suggested a mechanism of action of PRIMA-1^MET^ at least in part independent of TP53. PRIMA-1^MET^ was first isolated based on its specific action on TP53 mutated cell lines and its ability to re-induce P53 dependent pathway [8]. However, several studies have shown that PRIMA-1^MET^ can induce cell death even in tumor models with no mutation or deletion of TP53 [11, 19, 20] suggesting TP53-independent mechanisms of action. Recently, Sobhani et al. show that siRNA knockdown of P53 did not influence the PRIMA-1^MET^-induced cell death in Waldenström macroglobulinemia cells [21]. Our results are in perfect agreement with those of Sobhani et al. [21] and confirm that PRIMA-1^MET^ might involve more complex mechanisms than those originally described [10] not restricted to a P53 transcriptional related activity [22].

The second striking result was that PRIMA-1^MET^ did not induce significant caspase-dependent-apoptosis in STS. Indeed, contrary to that observed in other tumor models [13, 22, 23], we did not found any caspase-3 activation when STS cells were treated with PRIMA-1^MET^. It was therefore apparent that the PRIMA-1^MET^-induced cell death in STS was following a caspase-independent pathway.

Oxidative stress is a common form of cellular stress and refers to an imbalance of the production and clearance of cellular oxidants resulting in high concentration of ROS and oxidative damage. Due to their high reactive potential in reducing-oxidizing reactions, ROS can oxidize lipids, proteins, and nucleic acids, which in turn compromise the normal cellular functions of these macromolecules leading to growth arrest, cell senescence, and even cell death [24]. Because PRIMA-1^MET^ was reported to induce ROS [10, 16, 17], we assessed endogenous ROS production, in our panel of STS cell lines. Indeed, we found an increase of ROS production in STS cells treated with PRIMA-1^MET^, irrespective of the presence of TP53 and this increase was inhibited by NAC. The addition of NAC counteracted cell growth inhibition induced by PRIMA-1^MET^ in our STS cell lines. Altogether, these results indicate that ROS induction play a crucial role in PRIMA-1^MET^-induced cell death in STS as reported in other tumor models [16, 17].

Mechanisms involved in cell death resulting from ROS induction are not fully elucidated yet. However, in vitro studies from mammalian cells suggested that ROS regulate autophagy in various cell lines as exogenous oxidative stressors induce autophagy [25]. By assessing LC3-II levels, we showed here that treatment with PRIMA-1^MET^ induced autophagy in STS cells. We observed this effect in two STS cell lines with mutated TP53 and in the wild-type and the mutated colon carcinoma cell lines. This suggests that induction of autophagy by PRIMA-1^MET^ is at least in part independent of the mutational status of TP53. Our results are in agreement with those reported by Russo et al. [26] indicating that PRIMA-1^MET^ can trigger autophagy in cancer cells regardless of the presence of TP53 and identified autophagy as a potential crucial pathway leading to STS cell death induced by PRIMA-1^MET^. Since relation between autophagy and cell death is still controversial [27], this will require further investigation.

The JNK pathway is a well-conserved mitogen-activated protein kinase (MAPK). JNK was first identified as a stress-activated protein kinase (SAPK) that responds to various stresses in mammalian cells [28]. It has been known for a long time that JNK can be activated by exogenous ROS. Moreover, data from cell culture substantiates the notion that JNK activated by oxidative stress jeopardizes cell survival as a pro-death signal [29]. Li et al. reported that PRIMA-1^MET^ induced cell death through the JNK pathway in a p53-dependent manner in colorectal cell lines [30]. Interestingly, we found here that PRIMA-1^MET^ induced JNK activation in IB130 (mutated TP53) and IB139 (wild-type TP53) indicating that this process was at least in part independent of TP53. Moreover, JNK activation was not present in STS cell lines for which PRIMA-1^MET^ induced autophagy. This may suggest that autophagy induction and JNK activation may represent alternative mechanism of STS cell death resulting from oxidative stress induced by PRIMA-1^MET^. Our experimental conditions applied on colon carcinoma cell lines equally shown that cell death was not caspase dependent and that PRIMA-1^MET^ toxicity is not dependent of TP53 status but largely involve oxidative stress.

## Conclusion

In conclusion, our results confirm that the mechanisms of action of PRIMA-1^MET^ are more complex than firstly described. We demonstrate here that PRIMA-1^MET^ anti-tumor activity in STS is P53-independent and results mainly from off-target effects involving ROS-associated toxicity without significant induction of caspase-dependent-apoptosis. For this reason, PRIMA-1^MET^ does not deserve further development as a TP53-targeted therapy in STS. However, further studies may be warranted to assess potential synergy of PRIMA-1^MET^ with recognized active drugs in STS that interfere also with the ROS pathway such as doxorubicin [31].

## Abbreviations

DAPI: 4’,6-diamidino-2-phenylindole
DCFDA: 2',7'-Dichlorofluorescin diacetate
DNA: Deoxyribonucleic acid
FACS: Fluorescent Activating Cell Sorting
MDM2: Mouse double minute 2
MAPK: Mitogen-activated protein kinase
MMP: Mitochondrial membrane potential
MT: Mutated
MTT: Methyl Thiazolyl Tétrazolium
NAC: N-acetyl cysteine
NCI: National Cancer Institute
NT: Not treated
p-JNK: Phosphorylated JNK
PBS: Phosphate buffered saline
PVDF: Polyvinyl Difluoride
RIPA: Radio-ImmunoPrecipitation Assay
ROS: Reactive oxygen species
SEM: Standard error of the mean
SAPK: Stress-activated protein kinase
STS: Soft-tissue sarcomas
TMRM: Tetramethylrhodamine Methyl Ester
WT: Wild-type
